# Coffee Consumption and Colon Cancer Risk: A Meta-Epidemiological Study of Asian Cohort Studies

**DOI:** 10.31557/APJCP.2020.21.5.1177

**Published:** 2020-05

**Authors:** Jong-Myon Bae

**Affiliations:** *Department of Preventive Medicine, Jeju National University College of Medicine, Jeju, Republic of Korea. *

**Keywords:** Coffee, colonic neoplasm, systematic review, meta-analysis

## Abstract

**Objective:**

A systematic review reported that coffee consumption would decrease risk of colon cancer in Asian women. But the systematic review arises the issue of duplication, so that a meta-epidemiological study was conducted.

**Methods:**

The selection criteria were defined that a prospective cohort follow-up study conducted to evaluate coffee consumption and risk of colon cancer in Asian and showed adjusted relative risk and its 95% confidence interval. In order to conduct meta-analysis, the highest versus lowest method was applied to extract relative risk and its 95% confidence intervals of the highest category. Random effect model was applied if I-squared value was over 50%.

**Results:**

After avoiding duplication, 9 cohort data were selected for meta-analysis. The summary relative risk (and their 95% confidence intervals) [I-square value] were 0.90 (95% CI: 0.79-1.03) [0.0%] in men, and 0.64 (95% CI: 0.36-1.15) [65.9%] in women, respectively.

**Conclusions:**

The results suggest that coffee consumption is not associated with the risk of colon cancer in Asian men and women. The findings of this study are consistent with the results of two systematic reviews conducted under the same hypothesis and selection criteria. Additional epidemiological studies are needed for the inflection of colon cancer risk as the dose of coffee increases and the difference in the protective effect by sex.

## Introduction

Colorectal cancer ranks third in incidence and second in mortality worldwide (Bray et al., 2018). While dietary habits are known as major risk factors (Vargas and Thompson, 2012), it has been suggested in the hypothesis that the consumption of coffee might decrease colon cancer risk based on the anti-oxidants and anti-mutagens effects of coffee ingredients (Higdon and Frei, 2006).

The results of 4 systematic reviews on prospective cohort studies conducted to elucidate the hypothesis are as shown in Table 1 (Giovannucci, 1998; Li et al., 2013; Akter et al., 2016; Sartini et al., 2019). All except the colon cancer in Sartini et al. (2019) of 4 systematic reviews reported no statistical significance. However, the following two issues arise in Sartini et al., (2019), which most recently reported. First, the authors reported the upper limit of the confidence interval for colon cancer was 0.998 and emphasized the statistical significance. But, it was presented as 1.00 in the forest plot. Thus, it might be regarded as a marginally statistical significance and it should be interpreted carefully. Second, the articles selected for meta-analysis included not only original articles but also two special articles using pooled analysis that combined and analyzed databases from several cohorts (Zhang et al., 2010; Kashino et al., 2018). In particular, in the meta-analysis of Asian women showing statistical significance, authors included Kashino et al., (2018) having the results calculated from 8 Japanese cohort data. By including Kashino et al., (2018), three cohort databases (Lee et al., 2007; Naganuma et al., 2007; Yamada et al., 2014) were duplicated. In other words, there is a need to exclude duplication from the Sartini et al., (2019) and then conduct a new meta-analysis in Asians.

Therefore, the purpose of this study is to conduct a meta-epidemiological study (Bae, 2014) to re-evaluate the risk of colon cancer associated with coffee consumption in Asians.

## Materials and Methods

In that the previous systematic review was reevaluated, the selection criteria is the same as Sartini et al., (2019), but adds that it is limited to Asians. In other words, the selection criteria were defined that a prospective cohort follow-up study conducted to evaluate coffee consumption and risk of colon cancer in Asian and showed adjusted relative risk (RR) and its 95% confidence interval (95%CI). In response, five cohorts selected by Sartini et al., (2019) were subject to priority consideration (Oba et al., 2006; Lee et al., 2007; Naganuma et al., 2007; Yamada et al., 2014; Kashino et al., 2018). 

Meanwhile, the RR and its 95%CI was extracted using the ‘highest versus lowest method’ (HLM) in the selected papers as performed by Sartini et al. (2019). For each paper, logarithm RR (logRR) and standard error of logRR (SElogRR) were calculated from the information extracted by HLM method.

The heterogeneity level was evaluated by the I-squared value (%), and meta-analyses applying random effect model was performed in the case of more than 50% (Harris et al., 2008). Subgroup analysis was performed by sex. Statistical significance level was set as 0.05

## Results

Instead of selecting Kashino et al., (2018) in order to include eight Japanese cohort data, three papers (Lee et al., 2007; Naganuma et al., 2007; Yamada et al., 2014) were excluded to avoid duplication. As a result, 9 cohort data from two papers (Oba et al., 2006; Kashino et al, 2018) were selected for meta-analysis, finally. All were cohorts of Japanese population.

**Figure 1:**
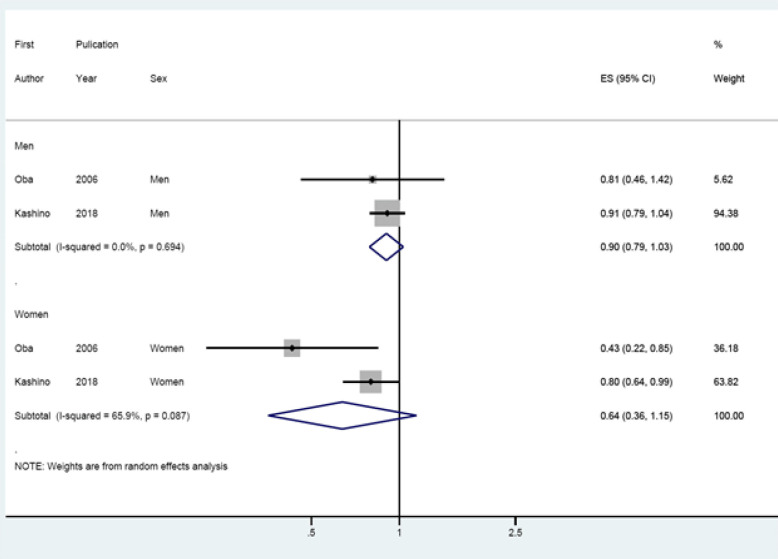
Forest Plot of Estimating Summary Effect Size (ES)

In the two final papers selected for meta-analysis (Oba et al., 2006; Kashino et al, 2018), the RR values extracted by applying the HLM method are presented in Figure 1. Both papers showed statistically significant protective effects in women. However, the meta-analysis from these results showed that the I-squared value was 65.9% and there was no statistical significance in women (sRR=0.64, 95% CI: 0.36-1.15).

**Table 1:** Summary Relative Risk (95% Confidence Intervals) [I-Squared Value] of Four Systematic Reviews for Population-Based Cohort Studies

FA (YP)	Giovannucci (1998)	Li et al., (2013)	Akter et al., (2016)	Sartini et al., (2019)
Search to	Jun 1997	May 2011	Aug 2015	-
Selected	5 CO	6 CO	5 CO (Japanese)	26 CO
Colorectum	0.97 (0.73-1.29)	0.94 (0.88-1.01) [0]	0.46 (0.18-1.16) [0.0]	0.96 (0.88-1.03)
Colon	-	0.93 (0.86-1.01) [0]	0.98 (0.70-1.36) [55.5]	0.91 (0.83-0.998) [53.4]
Rectum	-	0.98 (0.88-1.09) [0]	0.99 (0.72-1.37) [0.0]	1.00 (0.89-1.11)

CO, cohort study; FA, first author; PY, publication year; YP, year of publication

## Discussion

The results suggest that coffee consumption is not associated with the risk of colon cancer in Japanese men and women (sRR=0.64, 95% CI: 0.36-1.15). The conclusion differs from what Sartini et al. (2019) claimed to have statistically significant preventive effects in Asian women (sRR=0.73, 95% CI: 0.58-0.88).

This different result could be considered by two things. First, it is due to the duplication of cohort databases used in meta-analysis (Choi et al., 2014). As pointed out in the introduction, the results of this study was obtained after excluding three papers with duplication of cohort data. Second is the choice of effect model according to the level of heterogeneity. Since the I-squared value of this study was 65.9%, a random effect model was applied and the result was statistically insignificant. However, I-squared value of Sartini et al., (2019) was 43.1%, and authors stated clearly that a fixed effect model was applied. When this study ignored the heterogeneity level and applied the fixed effect model, a statistically significant result was obtained (sRR=0.75, 95% CI: 0.61-0.93). In other words, the elimination of duplication led to differences in the statistical significance of sRR as the level of heterogeneity increased.

The findings of this study are consistent with the results of two systematic reviews conducted under the same hypothesis and selection criteria (Akter et al., 2016; Horisaki et al., 2018). Although this study selected Kashino et al., (2018) instead of the three papers (Lee et al., 2007; Naganuma et al., 2007; Yamada et al., 2014) they had selected, but the same results were obtained. Of particular note, the pooled hazard ratios for the eight cohorts (Kashino et al., 2018) showed a significantly dose-response relationship with a P for trend of 0.05 when categorized into 1-2 cups or 3 cups or more, based on the intake group below 1 cup per day. In response, the dose-response meta-analysis (Horisaki et al., 2018) showed that the RR were lower than 1 as the daily intake increases from 1 cup to 3 cups, and then higher as more than 4 cups. These results indicated that there was a change of RR direction between 3 cups and 4 cups daily. Therefore, additional epidemiological studies are needed for the inflection of colon cancer risk as the dose of coffee increases and the difference in the protective effect by sex.
